# Urban Biomimicry for Flood Mitigation Using an Ecosystem Service Assessment Tool in Central Wellington, New Zealand

**DOI:** 10.3390/biomimetics8010009

**Published:** 2022-12-24

**Authors:** Maggie MacKinnon, Maibritt Pedersen Zari, Daniel K. Brown, Rubianca Benavidez, Bethanna Jackson

**Affiliations:** 1 Wellington School of Architecture, Wellington Faculty of Architecture and Design Innovation, Te Herenga Waka Victoria University of Wellington, Wellington 6011, New Zealand; 2 School of Future Environments, Faculty of Design and Creative Technologies, Te Wananga Aronui o Tamaki Makau Rau Auckland University of Technology, Auckland 1010, New Zealand; 3 School of Geography, Environment and Earth Sciences, Te Herenga Waka Victoria University of Wellington, Wellington 6011, New Zealand; 4 BEEA Limited, Wellington 6016, New Zealand

**Keywords:** urban biomimicry, ecosystem services, flood mitigation, green infrastructure, green roofs, simulation, urban design, climate change adaptation, nature-based solutions

## Abstract

Many cities are vulnerable to flooding due to their high proportion of impervious surfaces and lack of vegetated land cover. This vulnerability will often be exacerbated by changing rainfall and storm patterns due to climate change. Using the principles of urban biomimicry, this study aims to show an ecosystem service-based approach to designing an urban green infrastructure network for stormwater management in densely built areas that more closely emulates natural hydrology processes. Nature Braid (next-generation LUCI) is an ecosystem services assessment tool that was used to simulate flood mitigation ecosystem services in a 13.7 km^2^ urban water catchment in Wellington, Aotearoa New Zealand. The simulation results revealed that 59% of the catchment does not contain or benefit from flood-mitigating land cover features. Adding 0.6 km^2^ (4% of the catchment) of green roofs alongside major stormwater flow paths resulted in a nearly three-fold decrease (11%) in the unmitigated flooding area. These results suggest that green roofs could help manage stormwater and mitigate flooding in the densely built areas of the catchment. Using ecosystem service assessment tools, like Nature Braid, can inform the design of more regenerative and resilient urban green infrastructure networks that help mitigate climate change impacts on urban residents.

## 1. Introduction

As population growth and urbanisation drive the expansion of urban environments [1,2], the problem of urban flooding can have deleterious impacts on an increasing number of people and cost billions of dollars in damage [3]. Cao et al. [4] found that the global urban exposure to flooding was more than four times higher in 2018 than in 1985, attributed to cities expanding further into floodplains. Combined with the anticipated increase in the intensity and frequency of extreme rainfall events as the atmosphere warms [5], urban flooding risks will increase, particularly for cities in floodplains, on coasts, or next to waterways [6]. The dominance of buildings, grey infrastructures, and impervious surfaces in cities makes them less able to manage flooding than the pre-development sites, which typically possessed much more vegetated land cover and the capacity to absorb significant rainfall into the soil [7]. The large-scale disruption to natural hydrological processes in built environments has contributed to the issue of urban flooding, but emulating and restoring some of these processes could provide a solution. An ecosystem-level biomimicry approach, as described by Pedersen Zari [8,9], could improve urban stormwater management by regenerating critical flood mitigation ecosystem system services in cites. As a complex, dynamic network of organisms, processes, and habitats, ecosystems are one of the most suitable models for urban biomimicry strategies to begin to address and re-design the buildings, infrastructures, and green spaces in cities [10,11]. Though the numerous benefits, processes, and resources provided by ecosystems are difficult to accurately capture, particularly in urban settings, the ecosystem services model has become a popular way of understanding and discussing their value [12,13]. It will be used in this paper to discuss the ecosystem services, namely habitat provision and flood mitigation, that will be most critical in the development of an urban biomimicry strategy to address urban flooding.

Habitat provides the foundational abiotic and biotic resources, such as soil and plants, that support flood mitigation ecosystem services, such as rainfall absorption, evapotranspiration, and disturbance prevention [14]. Typically, the habitats that would have managed rainfall have been replaced in cities by grey infrastructures such as stormwater channels and underground pipes [15]. However, these offer fewer broad ecosystem benefits and are less able to adapt to changes in rainfall patterns and storm intensity as climate change progresses [16]. Chen et al. [17] found that grey stormwater management infrastructure has disadvantages, including insufficient pipe capacities and backwater flooding that can lead to local inundations. Green, blue, and hybrid infrastructures, like rain gardens, green roofs, and constructed wetlands, have been increasingly recognised in literature and policy as beneficial tools for urban flood mitigation [18]. Xu et al. [19] found that coupling grey infrastructure with green infrastructures can reduce flooding and have life cycle cost savings of up to 94%. Green roofs, in particular, could increase urban flood mitigation ecosystem services in densely built areas with little ground-level green space. Shafique et al. [20] found that green roofs could retain 10–60% of rainfall and slow runoff (depending on the duration and severity of the event), thereby reducing the risk of flash flooding. Zhang et al. [21] found that 81–85% of stormwater was retained for green roofs with 10 cm substrates and that there was an 82–210 min delay in runoff generation. In some instances, green roofs can also reduce contaminant loads in stormwater runoff [22]. While the benefits of green roofs for stormwater management have been well documented in the literature at the single-building scale, how to plan and implement them at a wider urban scale for strategic stormwater and flooding management has been less well studied.

Spatial multi-criteria analyses, such as the ones conducted by Venter et al. [23] and Langemeyer et al. [24], can provide valuable socio-cultural information for urban green infrastructure planning; however, they lack the more quantitative and iterative feedback that spatial modelling and simulation tools can provide planners and designers. Ecosystem service assessment tools use a variety of datasets (GIS, field surveys, interviews, remote sensing, public surveys, etc.) to classify and quantify the value of different ecosystem services [25]. While some produce outputs that include monetary values or social benefits, those that produce quantifiable biophysical outputs are particularly useful when designing built environments [26]. By modelling and simulating the complex interactions between ecosystems and cities, built environment professionals can better map and quantify the impacts of their projects on various ecosystem services, including habitat provision and flood mitigation, as well as identify specific spatial areas of priority for design interventions across whole cities or watersheds.

Predicting and managing flooding risk will be critical to the well-being and resilience of urban residents as climate change progresses. Despite a large field of literature on the benefits of green infrastructures, like green roofs, there remains a reliance on grey stormwater management infrastructure to manage flooding in urban environments. Better design and simulation tools that help optimize the design of urban green infrastructure networks and quantify their benefits could help increase the uptake and implementation of green infrastructures by local governments and landowners, thereby helping cities emulate ecosystem processes and functions more effectively. Using Wellington, New Zealand, as a case study, this research aims to show how an ecosystem service assessment tool, Nature Braid, can be used to guide the design of an urban green roof network to increase flood mitigation ecosystem services across a city. Nature Braid is one of the ecosystem service tools most suitable to Oceanic sites [25] and is currently being used in various applications by researchers at Te Herenga Waka Victoria University of Wellington [27], making it the most suitable tool for ecosystem service assessments in the study area.

## 2. Materials and Methods

### 2.1. Site

Located in the South Pacific on the North Island of Aotearoa New Zealand, Wellington is a temperate coastal city with a population of approximately 203,000 [28]. Wellington has a vested interest in its native ecosystems, as demonstrated by its membership in the Biophilic Cities Network [29] and key policy documents, such as the Our Natural Capital Biodiversity Strategy and Action Plan [30] and the Wellington Central City Green Network Plan [31]. Wellington’s development is constrained by the city’s hilly topography and its coastal position set around a harbour in the Cook Strait. The preservation of a series of interconnected green spaces and native habitat reserves contributes to the city having a high proportion of visible green and blue spaces. Blaschke et al. [32] found that there was 20 m^2^ of green space per capita in central Wellington, which is higher than the minimum 9 m^2^ recommended by the World Health Organization [33]. However, increasing population growth and densification will decrease the per capita amount of green space and increase the demand for urban ecosystem services [34].

**Figure 1:**
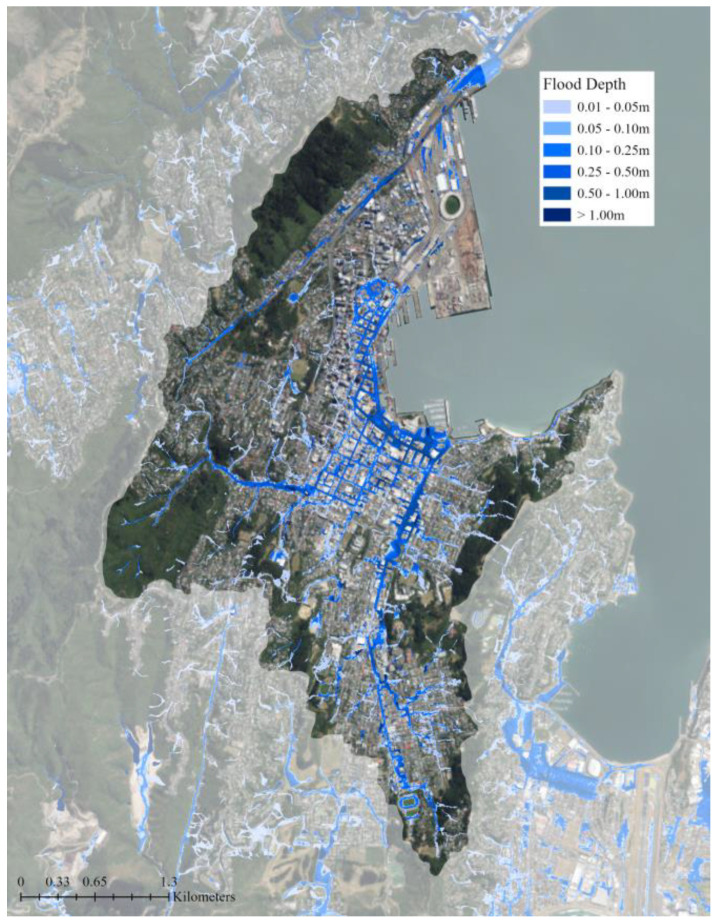
Flooding areas and depths in the Lambton Harbour-Oriental Bay catchment for a 1-in-100-year flooding event, taking into account climate change (assuming a 2.1 °C temperature increase) [42]. The worst flooding is predominately located along the coast and on major transportation arteries. The base aerial imagery was sourced from the LINZ Data Service [43].

The Lambton Harbour-Oriental Bay catchment (13.7 km^2^) (Figure 1), particularly, has a high density of existing buildings and is rapidly densifying to accommodate the city’s growing population. This catchment already has the lowest amount of green space in central Wellington, which is unlikely to be increased due to lack of space and development pressure [32,34]. McLean [35] found that 95% of the natural streams have been culverted beneath the city, removing the freshwater habitats and wetlands they would have naturally supported. It has also been identified by Capacity Infrastructure Services Ltd. (Lower Hutt, New Zealand) [36], the city council-controlled organisation that manages water infrastructures, as the highest priority catchment in Wellington for stormwater management retrofits due to its low-lying valley position and high proportion of impervious surfaces. Wellington City Council has a standing recommendation not to go swimming for 48 h after heavy rainfalls due to high contaminant loads in the waterways and coasts from runoff [37]. Heavy rains can also overwhelm the city’s existing stormwater management system, which resulted in more than 100 sewer overflows between April 2018 and March 2019 [38]. Based on data from 1981–2010, Central Wellington’s average annual rainfall is 957 mm [39]. Over the next several decades, there is anticipated to be a 10% rainfall increase (15% in winter) in the Wellington region [40]. Combined with increased storm intensity and frequency, this change in weather patterns will exacerbate the inadequacies in central Wellington’s existing stormwater infrastructure and result in more flooding [41]. As the building density in this neighbourhood increases, less ground-level green space will be available to mitigate increasing flooding vulnerabilities. However, the densification could be an opportunity to design new and retrofitted buildings with integrated green, blue, and hybrid infrastructures for stormwater management and climate change adaptation. With a few exceptions [23,24], there is limited research on urban-scale planning of green infrastructures that include buildings. Testing how these infrastructures could be designed in a densifying urban area is the aim of this research. Many different types of urban green, blue, and hybrid infrastructures could and should be used in a robust urban stormwater management network. For this paper, green roofs have been selected as the focus due to the limited ground-level space for green infrastructures in the study area.

### 2.2. Nature Braid

Nature Braid, the next generation of the Land Utilisation and Capability Indicator (LUCI), is a GIS-based framework and accompanying embedded tools that simulate the impacts of land cover and management on different ecosystem services and allows the user to compare where synergies and trade-offs occur [27]. Nature Braid includes ten ecosystem service models that are mature enough to be passed for application by external users: agricultural productivity, carbon stocks and fluxes, erosion and sediment, evaporative cooling, flood mitigation, habitat connectivity, habitat suitability, nitrogen, and phosphorus. An analysis by Delpy et al. [25] of different ecosystem services assessment tools found that Nature Braid was one of the tools most suited for cities in Oceania (of which Aotearoa New Zealand is part), and it produces outputs that both ecologists and urban designers can use. Nature Braid is particularly well suited to the Aotearoa New Zealand and Wellington contexts as tool development was led by researchers working in and around the Wellington region. It has been used for various research applications in Wellington and other parts of Aotearoa New Zealand for many years. It has also been used in Australia, the United Kingdom (where it first originated), the Philippines, Vietnam, and several Pacific Islands [27]. The tool can be applied to multiple scales, with a fine resolution of 5 m × 5 m recommended for most use cases. For urban contexts, if higher resolution data is available, 1 m × 1 m or 2 m × 2 m can also be used. Nature Braid is open source and publicly accessible by contacting the developers [27], as was done for this paper.

### 2.3. Test Model

**Figure 2:**
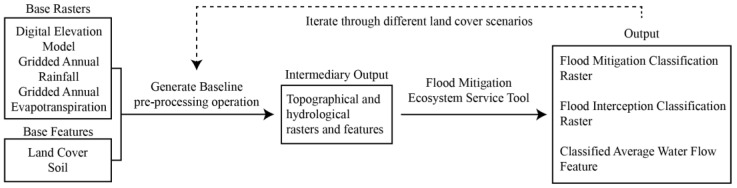
Nature Braid workflow. First, base raster and feature data were collated in GIS. The generate baseline pre-processing operation was run to generate intermediary topographical and hydrological data. These intermediary outputs were then used to run the individual ecosystem services tool for flood mitigation. The flood mitigation ecosystem service tool generated flood mitigation classification, flood interception classification, and average waterflow data, which can be used to inform future iterations of land use scenarios [51].

Nature Braid requires a set of input data, including digital elevation, land cover, soil, and climate data (Figure 2). The Wellington City LiDAR 1 m DEM (2019–2020) was obtained from Land Information New Zealand [44]; however, it only mapped the elevation of the ground plain and left out all buildings. The Wellington Buildings shapefile [45] was used to add building height information to the base DEM. Though some building footprint polygons contained height information, a large portion of the more than 15,000 polygons did not. Building footprint polygons were organised into Zones based on the Wellington City Council District Plan [46] to expedite the data entry process, and the maximum building heights for each Zone were applied. The Raster Calculator function of ArcGIS Pro was then used to combine the DEM raster with a building height raster generated from the building footprint polygons. With this approach, all building roofs are represented as flat in the final DEM raster, though realistically, there would be more variation in roof shape and slope. While this may be less important for ground-level green infrastructure, it could impact the design and testing of building-integrated green infrastructures, like green roofs. The land cover data was obtained from the national Land Cover Database [47] and was cross-checked with satellite imagery and fieldwork. One significant green space, Waitangi Park, had been classified as ‘Built Area’ in the original file, and this was corrected to the ‘Urban Parkland/Open Space’ classification. The national Fundamental Soil Layers North Island [48] dataset was used; however, the data it provided was limited due to most of the catchment being classified as ‘town’ with no differentiation between the soil in most urban parks and the soil under buildings. Default national values within Nature Braid can be used for rainfall and evaporation; however, gridded average annual rainfall and evaporation rasters [49] for the study area were used to increase the accuracy of the simulations. The model’s hydrological routing can also be improved by inputting a stream network shapefile. However, the national stream network data [50] was not accurate for the study area because streams are almost completely culverted under the city, so the ‘generate stream network directly from DEM’ option was used to simulate how buildings and topography affect overland surface flow.

### 2.4. Flood Mitigation Ecosystem Service Tool

The outputs generated from pre-processing were then used in the flood mitigation ecosystem service tool. This tool uses soil permeability and land cover to generate maps of features that help mitigate flooding and high stream flow and where overland stormwater flow accumulates [51]. Features with higher water storage and infiltration capacities, such as forests and wetlands, reduce and slow overland stormwater flows and mitigate flooding downstream. Built areas and transportation infrastructure have low permeability or storage for stormwater, resulting in unmitigated overland flow. The tool analyses these features in the study area to determine priority areas for improving stormwater management.

**Figure 3:**
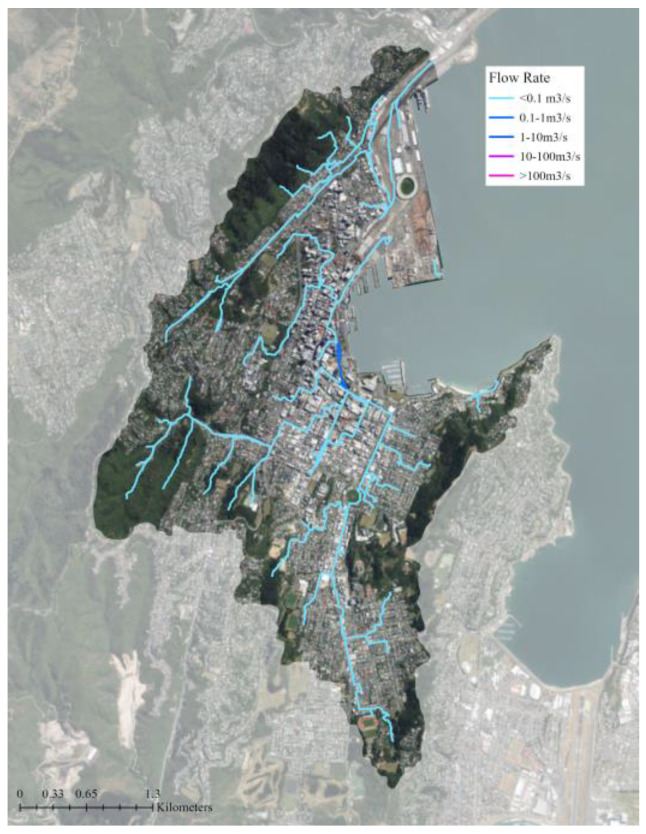
The average water flow class output from the flood mitigation tool. The blue lines show major overland stormwater flow paths and flow rates (m^3^/s). The base aerial imagery was sourced from the LINZ Data Service [43].

**Figure 4:**
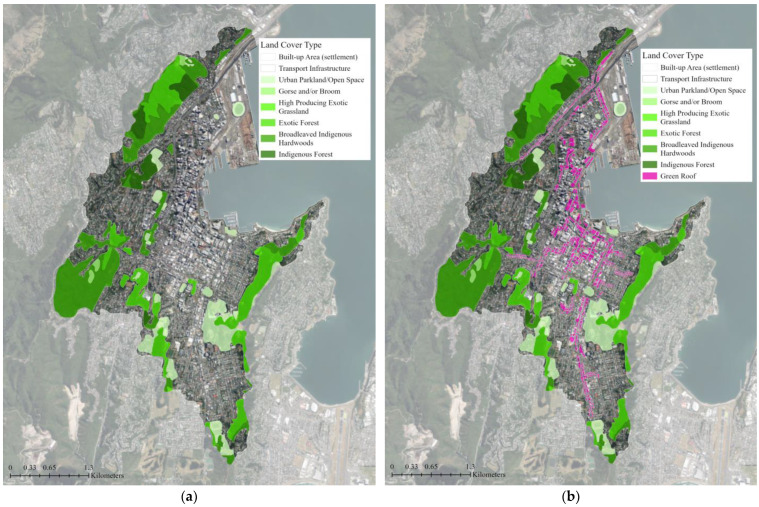
Land cover classification maps for: (**a**) The existing green infrastructure network; (**b**) The proposed green infrastructure network with supplemental green roofs. The base aerial imagery was sourced from the LINZ Data Service [43].

The flood mitigation tool uses the model’s spatially explicit representation of topography, land cover, soil, and other characteristics to understand how flow moves through the landscape (i.e., whether it is being mitigated or not or if it provides mitigation benefits). Nature Braid is able to represent overland and subsurface flow based on topography, land cover, and soil, but does not yet include subsurface flow due to grey infrastructures, such as sewers and stormwater drains. However, its output remains useful (particularly in Wellington, where it is already known that the grey stormwater infrastructure is insufficient) as it identifies where stormwater flows and flooding most need to be managed, whether that be through green, grey, or hybrid infrastructures. The average water flow paths highlight areas where overland flows are most significant and where more mitigation is needed to slow their flow. These flow paths were used to guide the design of a green roof network that could help manage stormwater in the catchment (Figure 3). The building polygons were used to change the land cover classification (i.e., material composition) of 948 building roofs on either side of major flow paths. In total, 583,600 m^2^ (4% of the catchment) of green roof area was added to the base land cover file (Figure 4). The land cover classification ‘Urban Parkland/Open Space’ includes grassy or sparsely-treed open areas for recreation, amenity, or utility, such as playgrounds, parks, sports fields, and gardens [47]. This classification was selected for the green roofs as it was the most appropriate category to represent the designed planting communities of typical green roofs that are often similar to meadows or gardens and rarely contain large trees due to structural limitations [52]. In the green roof scenario, the total ‘Urban Parkland/Open Space’ land cover area was increased to 1.35 km^2^ from the existing 0.77 km^2^. The soil data was not altered for the green roof scenario because of the lack of soil data for the study area. Due to their elevation from the ground, the hydrology of the green roofs will be different than other green spaces in the area as they will only catch rainfall that falls directly on them. The Nature Braid generate baseline pre-processing and flood mitigation tools were run again with the new land cover data to see how adding green roofs impacted the flood mitigation outputs.

## 3. Results

**Figure 5:**
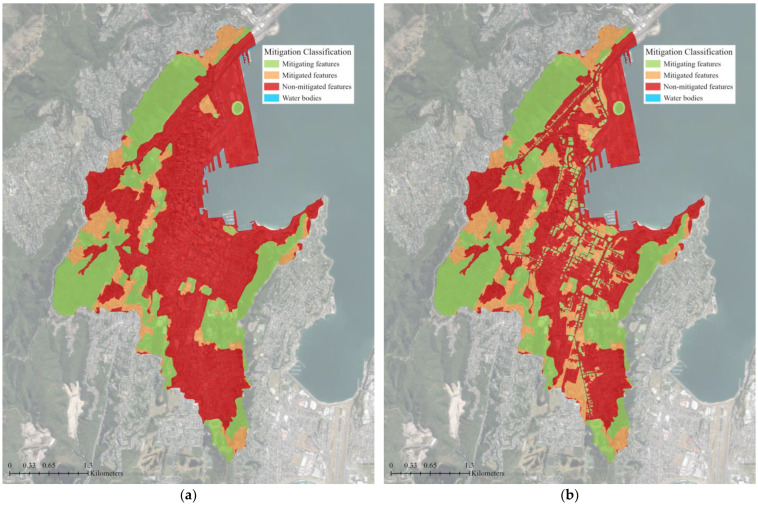
Nature Braid flood mitigation class map for: (**a**) The existing green infrastructure network; (**b**) The proposed green infrastructure network with supplemental green roofs. Flow mitigation features, such as green spaces or green roofs, are shown in green. Orange represents areas that benefit from the reduction and slowing of overland flows provided by the mitigating features. The majority of the catchment, shown in red, does not contain or benefit from mitigating features. The base aerial imagery was sourced from the LINZ Data Service [43].

**Figure 6:**
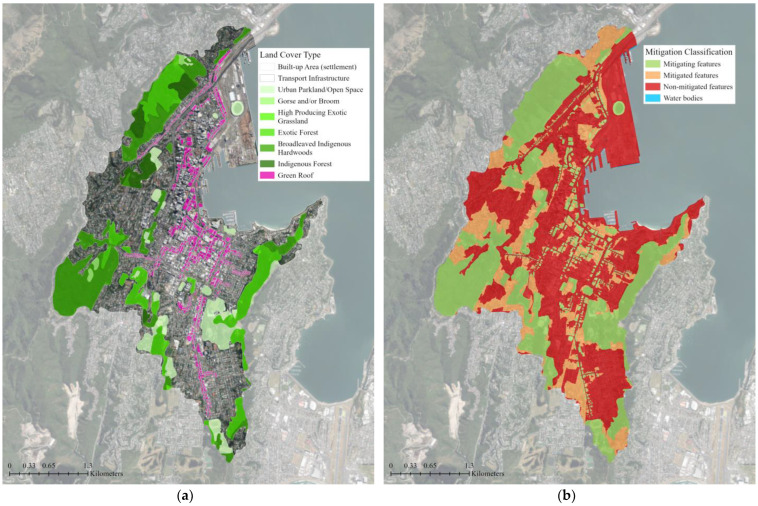
Input and output files for the Nature Braid flood mitigation tool: (**a**) The input land cover data for the proposed green infrastructure network with supplemental green roofs; (**b**) The flood mitigation classification output. The tool identified green roofs as flow mitigation features (shown in green) and, combined with the elevation data, determined how they could reduce and slow overland flows in their vicinity (shown in orange). The majority of the catchment still does not contain or benefit from mitigating features (shown in red); however, this area has been reduced by 11% with the addition of green roofs. The base aerial imagery was sourced from the LINZ Data Service [43].

The water accumulation thresholds for flood mitigation classifications are determined using a multiplier of the area. The default value of 5 was used, meaning an area that can accumulate and store flow more than 5 times its area is considered a mitigating feature [51]. With the existing green infrastructure in the study area, the flood mitigation class maps show that 30% of the study area is covered by mitigating features and an additional 11% of the study area benefits from the flood mitigation of these features (Figure 5). The majority of the study area (59%) does not contain or benefit from any mitigating features. When supplemental green roofs were added to the existing green infrastructure network, the area covered by mitigating features increased by 4% and the mitigated area that benefits from these mitigating features increased by 7% (Table 1). The ratio of mitigated area per mitigating area rose slightly from 0.4:1 to 0.5:1 for the green roof scenario. The addition of 0.58 km^2^ or 4% of mitigating features area (green roofs) resulted in a nearly three-fold decrease (1.49 km^2^ or 11%) in the non-mitigated flood area (Figure 6).

**Figure 7:**
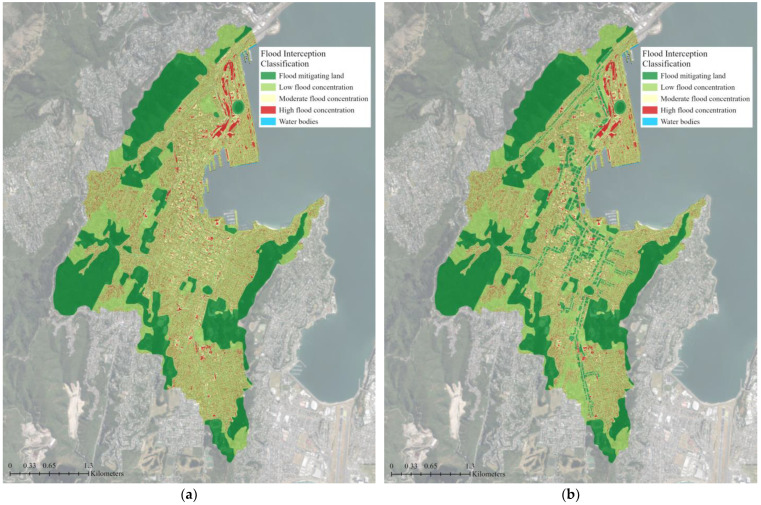
Nature Braid flood interception classification map for: (**a**) The existing green infrastructure network; (**b**) The proposed green infrastructure network with supplemental green roofs. Flood-mitigating land, such as green spaces and green roofs, is shown in dark green. Areas that most benefit from these features and have low flood concentrations are shown in pale green. Areas with moderate flood concentrations are shown in yellow, and high flood concentration areas are shown in red. These areas are the ones that could most benefit from features to improve water storage and slowing capacities to reduce flooding. The base aerial imagery was sourced from the LINZ Data Service [43].

**Figure 8:**
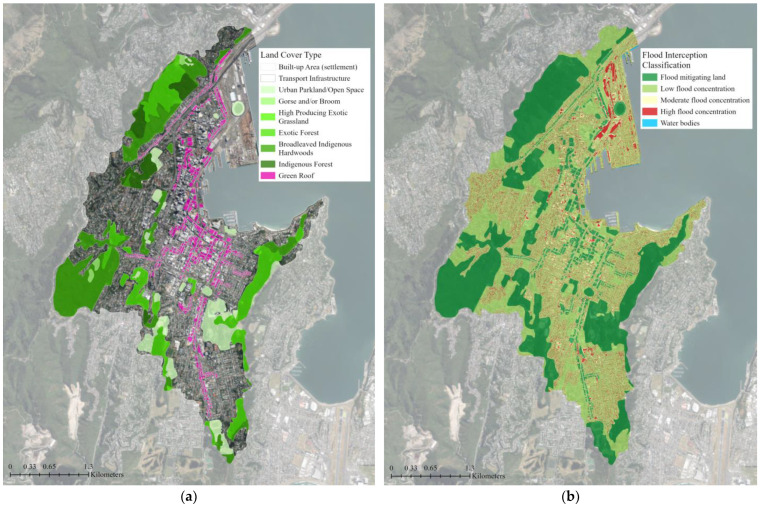
Input and output files for the Nature Braid flood mitigation tool: (**a**) The input land cover data for the proposed green infrastructure network with supplemental green roofs; (**b**) The flood interception classification map output. The tool identified green roofs as flood-mitigating land (shown in dark green) and, combined with the elevation data, determined how they could reduce flood concentrations. The green roofs did not contribute to any change in low-flood concentration areas (shown in pale green); however, they did reduce moderate (shown in yellow) and high (shown in red) flood concentration areas by 2% each. The base aerial imagery was sourced from the LINZ Data Service [43].

The flood interception classification maps can help identify the highest priority areas within the catchment for flood mitigation interventions [51]. They show that 30% of the study area is covered by flood-mitigating land, such as trees and deep permeable soils that slow and absorb rainfall (Figure 7). Low and moderate flood concentrations, where stormwater flow accumulations are somewhat mitigated by surrounding mitigating land, cover 50% and 10% of the catchment, respectively (Table 2). High flood concentration areas, where stormwater flows accumulate without mitigation, cover 10% of the catchment and are predominately located near the coast and around major transportation infrastructure. With supplemental green roofs, the area covered by flood-mitigating land increased by 4%, but there was no change to the low flood concentration area (Figure 8). The moderate flood concentration and high flood concentration areas decreased by 2% each.

## 4. Discussion

**Figure 9:**
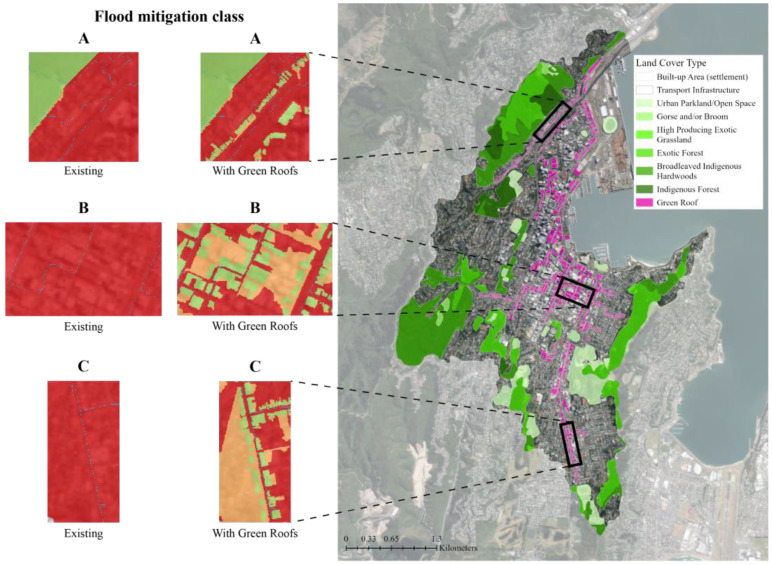
Optimising green roof locations for flood mitigation. Red represents non-mitigated features, orange represents mitigated features, and green represents mitigating features in the flood mitigation class maps. The small-scale, linear green roofs in area A do not provide as much flood mitigation as the larger, more clustered green roofs in areas B and C. This indicates that the location, size, and configuration of green roofs (mitigating features) impact how beneficial they are to stormwater management beyond their sites. The base aerial imagery was sourced from the LINZ Data Service [43].

The results of the Nature Braid simulation demonstrate that the proposed green roof network can aid in stormwater management in a densely built urban catchment. Though previous research has evidenced the stormwater management benefits of green roofs on a single building [20,21,22], this study shows how they can benefit an urban-scale stormwater management strategy. The 0.58 km^2^ of green roof area added to the Nature Braid simulation resulted in a nearly three-fold decrease (1.59 km^2^) in non-mitigated flood areas in the catchment, demonstrating the positive impacts they can have for flood mitigation beyond their individual sites. While these are promising results, there is still room to improve the design of the green roof network. As highlighted by area A in Figure 9, a linear series of small residential green roofs contributed little to increasing the mitigated area (shown in orange) around them and, therefore, may not be worth including in the green roof network. In comparison, the larger, clustered green roofs in area B increased the mitigated features area for their whole city block. The green roofs in area C, though relatively small and linear, resulted in a large increase of mitigated features area compared to area A, which may be due to their higher position in the catchment where the stormwater flows originate. This indicates that the location, size, and configuration of green roofs can significantly impact their value to stormwater management in their local area and potentially further downstream in catchments. Further iterations of the green roof network are needed to optimise the location and size of green roofs to ensure they provide the maximum benefit to stormwater management in the area.

While the Nature Braid tool can estimate the supply and spatial extent of ecosystem services in a given area, some important limitations must be mentioned. Gaps or inaccuracies in the base or input data can result in potential errors while running the tools or in the outputs produced. The LiDAR information for buildings was unavailable, and though a workaround was achieved using building footprints and heights, the DEM input could be improved by filling these gaps. This will be particularly important for refining the building selection for green roof networks, as roof shape and slope will determine their suitability for green roofs. Many other factors will also impact buildings’ suitability for green roofs, such as structural capacity, building code restrictions, and costs. These are beyond the scope of this analysis but could be done in future research to enhance the feasibility of the proposed green roof network. The information to supplement or amend gaps in the soil data was limited. The majority of the catchment was classified as the soil type ‘town’ with no differentiation between the soil classification of densely built areas and significant green spaces (such as the Botanical Gardens). More data on the soil types and permeabilities of the green spaces in the study area, as well as determining an appropriate soil classification for green roofs, would help improve the accuracy of the Nature Braid results and other similar biophysical and geospatial analyses. Though the primary focus of this study was to determine the amount and location of green roofs needed to reduce flooding in the study area, green roof type (extensive, semi-intensive, and intensive) impacts how they manage stormwater [53,54]. The ‘Urban Parkland/Open Space’ land cover classification was used in this study as a proxy for green roofs; however, more specific land cover classifications for green roofs based on their soil depths and planting communities would improve the accuracy of the results for stormwater retention and evapotranspiration [55]. Though the land cover file was altered to include green roofs, the gridded annual evaporation raster could not be altered in a similar way, which may have resulted in some inaccuracies. Nature Braid cannot currently simulate storm conditions as it requires an annual average rainfall input. A simulation was run with a 10% increased gridded annual rainfall raster based on climate change predictions for the study area. However, the results were deemed inaccurate because the 10% increase is predicted to occur only in certain seasons and in more severe storms, which could not be captured in the current version of Nature Braid. Despite these limitations, the tool can provide helpful information and guidance on priority areas for flood mitigation and estimate some benefits of adding additional mitigating features, such as green roofs, to targeted, specific locations. The tool helps identify which urban neighbourhoods, blocks, or streets should prioritise and incentivise green roof installation on new or existing buildings to improve stormwater management in a city.

Though this is the first study using the Nature Braid tool to design and simulate an urban green roof network, similar stormwater management benefits of widespread urban green roofs have been found using other modelling and simulation tools. Mora-Melià et al. [56] used the Storm Water Management Model software in Curicó, Chile to determine that the addition of 50% green roof coverage could prevent flooding for moderate rainfall; however, the authors found that 60–95% green roof coverage is needed to prevent flooding during stronger rainfall events. Ercolani et al. [57] used a distributed hydrologic model to assess the impact of green roofs during storms and found that the green roofs reduced both peak flow and volume in the urban stormwater management system. Twohig et al. [58] used the Arc-Malstrom model to identify high flood-risk areas in Helsinki and test several green roof scenarios. They found that the average flood depth could be reduced by up to 13% depending on the level of green roofs implemented. A major benefit of using green instead of grey infrastructures is the multiplicity of benefits they provide due to the synergistic relationship between habitat provision and many other ecosystem services. Using the environmental and urban planning simulation software ENVI-Met, Viecco et al. [59] found that the addition of green roofs and green walls to the downtown area of Santiago, Chile could reduce air particulate matter by up to 7.3% and help address the common issue of air quality in cities. They can also help reduce the urban heat island effect [60] and increase habitat connectivity for biodiversity [61].

This paper focused on the flood mitigation ecosystem service tool of Nature Braid; however, there are several other ecosystem service tools (erosion and sediment, habitat connectivity, etc.) that could be used in future research to develop an urban green infrastructure design that optimises the outputs for multiple ecosystem services, responds to additional climate change impacts, or addresses other societal challenges. The applications of Nature Braid and other ecosystem system service assessment tools are relevant to many disciplines. As has been demonstrated in this paper, they can help urban designers and planners map risk areas and design urban green/grey infrastructures to mitigate them. The tools can also help policymakers identify and draft suitable regulations and incentives to support urban-scale biomimicry projects. However, the successful implementation of these projects will depend upon knowledge from other fields. Architects and engineers can help determine the suitability and capacity of buildings and existing grey infrastructure to support green infrastructures, like green roofs. Local ecologists will also need to be consulted regarding the target species and habitats that green infrastructures should emulate, conserve, restore, or regenerate. Interdisciplinary collaborations are essential to creating buildings and urban green infrastructures that contribute to the regeneration of urban ecosystem services and help cities adapt to climate change.

## 5. Conclusions

As cities expand and increase in density, and climate change drives more frequent and severe rainfall events, the need for urban ecosystem services increases in quantity and urgency. This research found that large-scale implementation of green roofs (4% of the catchment area) resulted in a three-fold decrease (11% of the catchment area) in the unmitigated flood risk area. Urban biomimicry strategies and tools, like Nature Braid, can help planners, designers, policymakers, and stakeholders create more resilient urban green infrastructure networks that mimic natural hydrological processes and increase flood mitigation. Robust urban green infrastructure networks that increase flood mitigation and other ecosystem services, like habitat provision, air purification, and evaporative cooling, will be critical to the health, well-being, and resilience of urban residents as climate change progresses.

## Figures and Tables

**Table 1:** Quantitative results from the flood mitigation class output for the existing green infrastructure and supplemental green roof scenarios in the 13.7 km^2^ Lambton-Harbour Oriental Bay catchment.

Classification	Existing Scenario (km^2^)	Percentage of Catchment	Green Roof Scenario (km^2^)	Percentage of Catchment	Change in Area (km^2^)	Percentage Change in Area
Non-mitigated Features	8.05	59%	6.57	48%	−1.49	−11%
Mitigated Features	1.51	11%	2.42	18%	+0.91	+7%
Mitigating Features	4.07	30%	4.65	34%	+0.58	+4%

**Table 2:** Quantitative results from the flood interceptions classification output for the existing green infrastructure and supplemental green roof scenarios in the 13.7 km^2^ Lambton-Harbour Oriental Bay catchment.

Classification	Existing Scenario (km^2^)	Percentage of Catchment	Green Roof Scenario (km^2^)	Percentage of Catchment	Change in Area (km^2^)	Percentage Change in Area
Flood-Mitigating Land	4.06	30%	4.65	34%	+0.58	+4%
Low Flood Concentration	6.80	50%	6.76	50%	−0.04	0%
Moderate Flood Concentration	1.40	10%	1.11	8%	−0.29	−2%
High Flood Concentration	1.35	10%	1.10	8%	−0.25	−2%

## Data Availability

Some of the data presented in this study are openly available at [42,43,44,45,46,47,48,49,50].
